# Training medical educators to teach: bridging the gap between perception and reality

**DOI:** 10.1186/s13584-021-00509-2

**Published:** 2021-12-16

**Authors:** Alison Trainor, Jeremy B. Richards

**Affiliations:** 1 Division of Pulmonary, Critical Care, and Sleep Medicine, Beth Israel Deaconess Medical Center, 330 Brookline Avenue, KS-B23, Boston, MA 02215 USA; 2 Harvard Medical School, Boston, MA USA

**Keywords:** Continuing medical education, Faculty development, Teaching skills, Clinician educator, Medical education, Assessment

## Abstract

Teaching is a core expectation of physicians in academic hospitals and academic medical centers, but best practices for training physicians to teach have not been established. There is significant variability in how physicians are trained to teach medical students and residents across the world, and between Israeli hospitals. In an article published earlier this year in the Israel Journal of Health Policy Research, Nothman and colleagues describe a survey of 245 Israeli physicians in departments of internal medicine, pediatrics, and obstetrics and gynecology, at four different faculties of medicine across Israel. The majority of Israeli physicians responding to this survey reported receiving minimal training to teach, with only 35% receiving any training focused on medical education skills, most (55%) receiving training of only 1–2 days duration. In addition, the physicians surveyed perceived their training as inadequate and not aligned with their self-perceived educational needs. Furthermore, the respondents felt strongly that “compensation and appreciation” for medical education was less than for those involved in research. Despite the general lack of training in teaching skills and the perception that teaching physicians are less valued than researchers, survey respondents rated themselves as highly confident in most domains of medical education. In this context, this commentary reviews the disconnect between the general perception that *all* physicians can and should engage in teaching in the clinical setting with the well-described observation that competence in medical education requires dedicated and longitudinal training. Leveraging best practices in curriculum design by aligning educational interventions for teaching physicians with their self-perceived needs is discussed, and models for dedicated faculty development strategies for teaching medical education skills to physicians are reviewed. Finally, the importance of and potential strategies for assessing teaching physicians’ effectiveness in Israel and elsewhere are considered as a means to address these physicians’ perception that they are not as valued as researchers. Understanding teaching physicians’ perspectives on and motivations for training medical students and residents is critical for supporting the frontline teaching faculty who educate future healthcare providers at the bedside in medical schools, hospitals, and academic medical centers in Israel and beyond.

In a recent issue of the Israel Journal of Health Policy Research, Simon Nothman and colleagues describe an important study demonstrating that formal training for teaching physicians is perceived to be inadequate [1]. The authors surveyed teaching physicians in the departments of internal medicine, pediatrics, and obstetrics and gynecology, asking them to respond to questions assessing their perceptions of and training in medical education for teaching medical students in the clinical setting. Survey respondents were physicians responsible for training medical students in in-hospital clerkships (“teaching physicians”), and not necessarily physicians with expertise in or a primary career focus on medical education (“medical educators”). The authors found that only approximately a third of responding physicians received *any* training in clinical teaching (35%), and those physicians perceived their training as inadequate and not aligned with their self-perceived needs. The respondents also felt that teaching students was inadequately valued by promotion committees and hospital leadership as compared to faculty who focused on research pursuits.

## Confidence in teaching abilities

Physicians who work in clinical settings in academic medical centers are generally expected to teach medical learners as a core component of their day-to-day work. Despite this expectation, however, minimal training in medical education knowledge and skills for teaching physicians occurs not just in Israel, but throughout the world [2]. One possible explanation for this disconnect between the expectation that all physicians teach in clinical settings and the paucity of training in teaching skills is the inaccurate perception that teaching is an innate ability that *any* physician can perform. This perception may arise from the lack of emphasis on teaching students and residents’ educational skills during undergraduate and graduate medical education training, but also stems from a lack of resources and faculty development opportunities to train teaching physicians on effective clinical educational practices. The lack of focus on training physicians about teaching skills, yet the expectation that teaching is a core responsibility of all physicians in the clinical setting, contributes to the perception that *anybody* can teach without being taught how to do so.

This gap between being trained to teach yet being confident in one’s teaching abilities is consistent with the results of the current study, which demonstrated that respondents felt their medical education training was inadequate, yet rated themselves as highly in confidence in most domains of medical education [1]. Other studies have also revealed that in general physicians rate themselves highly with regard to their teaching abilities, while residents and medical students rate those same physicians lower with regard to teaching skills [3–5]. Although self-assessment is known to have its limitations [6], highly self-rated confidence in one’s abilities, juxtaposed against the recognition that the training physicians receive about teaching skills is inadequate, highlights the deeply embedded perception that medical education is not a skill that requires rigorous training.

The distinction between teaching physicians and medical educators underscores these issues. Teaching physicians are clinicians who are primary responsible for training medical students and residents in the clinical setting, but in general have received minimal to no training on how to effectively teach trainees. Medical educators, in contrast, have generally received more extensive, dedicated training on pedagogy, educational theory, and potentially medical education research, and their career focus is on best practices in medical education. Nothman and colleagues studied the perceptions and attitudes of teaching physicians, not those of dedicated medical educators, which is appropriate, as the vast majority of medical students in Israel and beyond are taught by teaching physicians on in-hospital clinical rotations.

The distinction between teaching physicians and medical educators provides contrast in the preparation and training that these two different classes of academic physicians receive. As an example, the lack of recognition that being a successful and effective teaching physician requires dedicated training is highlighted by the observation that longitudinal medical education experiences, as opposed to time-limited workshops and short courses, improve physicians’ medical education knowledge and skills [7]. Despite improved outcomes attributable to longitudinal programs to train physicians to teach, they constitute a minority of the faculty development interventions that are offered to teaching physicians [7].

## Importance of dedicated, longitudinal training for teaching skills

The majority of the teaching physicians who responded to the current study report having received 0–2 days of training in medical education. Two days of training about basic science or clinical research skills would never be expected to result in research expertise, and it would be laughable to anticipate that two days of training in an area of clinical practice would result in even basic competence or readiness for independent practice. The same holds true for medical education: teaching physicians will not achieve competence (or certainly expertise) in medical education with only short, time-limited, sporadic faculty development experiences.

So, how can medical schools, academic medical centers, and other institutions in which teaching physicians are expected to teach bridge the gap between the perception of physicians having universally acceptable teaching skills and the reality of the need for more dedicated and focused training of teaching physicians? Several organizations have identified multiple core competencies for being a medical educator [8, 9], which include complex abilities and attributes such as interpersonal communication skills, practice-based reflection, and medical knowledge. These are not skills that can be developed through brief workshops or faculty development retreats. Rather, there is growing recognition that competence and expertise in medical education requires deliberate practice, which consists of longitudinal, repetitive practice with expert coaching, focused feedback, goal setting, and self-evaluation [10]. This recognition has prompted the development of more rigorous training programs for faculty in medical education [11, 12]. However, a minority of physicians responsible for teaching medical students, residents, and fellows undergo such training. In this context, we recommend that medical education skills should be taught early for all physicians, starting during undergraduate medical education, just as with research and clinical skills [13, 14]. For those who identify as medical educators, with medical education as their career focus, medical education fellowship programs can serve as a model of more intensive, focused, and longitudinal faculty development strategies to develop the skills necessary to be a leader in medical education [11, 12]. Medical educators, trained through such dedicated and immersive faculty development programs, can become important resources for training teaching physicians on best practices for clinical teaching, helping to bridge the gap between teaching physicians’ perception of competence and their actual teaching skills. The potential benefit of incorporating medical educators into faculty development for training teaching physicians has been repeatedly called for in the literature over the past 20 years [15, 16].

## Assessing physicians’ teaching skills

While there is growing recognition that acquiring the knowledge, skills, and behaviors to become an effective medical educator requires specific training, it remains unclear what are the optimal strategies to assess physicians’ medical education skills and their success (or opportunities for improvement) as medical educators or teaching physicians. In this context, Nothman and colleagues identify that teaching physicians lack adequate, specialized training, and respondents perceive that medical education is also seen as inferior to research by hospital leadership and promotion committees [1].

In addition to the perception that any physician can teach, the lack of recognition for training medical students and residents in academic medical centers may be due to the linear and predictable course of academic research careers, which have clearly defined milestones. For example, grant funding and publications are visible, quantitative, and valued, whereas success for clinician-educators is more difficult to define. The lack of clear metrics of success for teaching physicians makes evaluation and promotion more difficult, which further contributes to the sense of less recognition of, and appreciation for, medical education skills by hospital and medical school leadership. These considerations explain why the majority of respondents in the current study indicated that teaching is inadequately acknowledged and recognized... and these results are not specific to Israeli medical schools but reflect the perceptions of academic clinician educators around the world.

Given these considerations, it is imperative that efforts are made to develop ways to measure success in teaching in the clinical setting, as lack of recognition and delayed promotion can lead to faculty burnout and attrition [2, 17]. To address this issue, Nothman and colleagues’ study gives some indications as to what might be effective measures to evaluate teaching physicians based on the respondents’ perceptions of what are important motivations for teaching. The factors rated as most important by respondents regarding motivation to teach were understanding that teaching improves the physician’s learning, the physician’s desire to contribute to the next generation of healthcare professionals, and understanding that teaching is integral to the physician’s core identity. In response to an open-ended question regarding the most important aspects for training physicians to teach in the clinical environment, three broad themes emerged: systemic factors, reward and recognition, and plans with clear goals and objectives [1]. While not clearly defined by Nothman and colleagues, the phrase ‘systemic factors’ indicates resources and support from the physician’s organization, institution, and/or environment, things that are not directly under the individual physician’s control. Of note, physicians who are more experienced at training learners were more likely to cite systemic factors as being important for medical education in the clinical environment, as compared to less experienced training physicians, who were more likely to identify guidance in understanding expectations, developing teaching plans, and implementing teaching methods.

In order to make transparent, well-informed decisions about promotion and career advancement, valid and reliable measures need to be identified to assess how effectively educators are educating and training learners and how they are contributing to local, regional, national, and international medical education practices. An ultimate, though difficult to attain, goal in medical education is to identify if and how medical education interventions improve patient care. While this should remain an objective of assessing medical education interventions and core medical educators’ skills, there are other more proximal, achievable, and measurable factors that can be used to assess teaching physicians and medical educators. These other factors include, but should not be limited to, trainee feedback. Other parameters that should be incorporated into formal assessments of core medical educators’ effectiveness include peer feedback, impact of medical education materials on websites such as MedEdPORTAL the quantity and type of educational sessions led, national and international conference presentations, curriculum development, and medical education scholarly publications, among others (Table 1). Determining which metrics are pertinent for medical educators as compared to teaching physicians is critical, to ensure that the assessments of these different types of training physicians align with performance expectations. Specifically, trainee and peer evaluations may be more heavily considered for teaching physicians, while scholarly productivity as evidenced by submissions to peer-reviewed journals may be more important for medical educators. One way to comprehensively review these metrics is by incorporating these other factors into a teaching portfolio for means of evaluation and promotion [18, 19].

**Table 1:** Strategies for assessing teaching physicians and medical educators, with specific examples

Means of assessing teaching physicians and/or medical educators	Examples
Trainee feedback	Quantitative and qualitative feedback from teaching sessions and/or clinical rotations
Peer feedback	Dedicated peer observations of teaching in formal (e.g., conference room-based teaching) and informal (e.g., on rounds) teaching settings, potentially using pre-existing teaching observation checklists
Impact of peer reviewed medical education content	Number of citations and/or number of views and/or downloads of open access articles on sites such as MedEdPORTAL (https://www.mededportal.org/)
Quantity and type of educational sessions led	Teaching sessions for medical students, residents, and other learners can be delineated, described, and codified by the medical educator for review
National and international conference presentations	Presentations at professional society meetings and/or at other institutions (in-person or remotely)
Curriculum development	Courses, teaching sessions, and/or curricula developed by the medical educator can be delineated with accompanying teaching materials, as a component of the educator’s teaching portfolio
Medical education scholarly peer-reviewed publications	Publications in standard, peer-reviewed journals
Non-peer-reviewed educational products	Educational resources that have been created and disseminated through social media, blogs, the educator’s own website, or other venues
Participation and leadership in professional societies	Membership, engagement, and specific activities, roles, and/or presentations in the context of specific professional societies
Reviewer and/or editorial roles in peer-reviewed journals	Service and leadership roles in journals
Medical education research	Funded or unfunded research focused on medical education interventions, assessments, and/or evaluations, even if not completed or published

## Conclusion

Nothman and colleagues are to be commended for their work demonstrating that a cohort of Israeli teaching physicians have a desire to participate in educational experiences with the goal of improving their medical education knowledge and skills. Furthermore, that lack of recognition and inadequate support are the main self-perceived barriers to success as teaching physicians identified in this study highlight specific areas medical school and hospital leadership can focus on to improve support of both teaching physicians and medical educators. In addition, while there have been calls to action for decades to integrate medical educators into the structure of academic medicine and specifically into training teaching physicians on best practices for teaching in the clinical setting, there remains significant room for improvement. While the value and necessity of teaching physicians in medical schools, hospitals, and academic medical centers is not in question, Nothman and colleagues’ study emphasizes that more work is needed to identify concrete and effective means by which to train, maintain, and promote the physicians who train medical students and residents.

It is important to recognize that although some training is better than no training, workshops and brief courses are insufficient to equip teaching physicians with the skills they need to become competent or expert clinician educators. Existing medical student curricula, clinical educator tracks in residency education, and post-graduate medical education fellowships can serve as models for training future and current clinician educators, and actively leveraging medical educators to train teaching physicians can help to bridge this gap. Furthermore, training alone is not enough to support and maintain the knowledge, skills, and motivation of teaching physicians and medical educators who are integral to academic medical centers. Similar to how research and clinical expertise are evaluated, medical education should be consistently and thoughtfully evaluated with the goal of equitable, transparent, and reproducible recognition and promotion processes for all.

## Data Availability

Not applicable.
